# The National Pension Fund

**Published:** 1887-07-02

**Authors:** 


### THE NATIONAL PENSION FUND.

IT will save a good deal of trouble if nurses and other hospital officials who propose joining the National Pension Fund will send their names and other particulars to *the Secretary of the Hospitals Association*, Norfolk House, Norfolk Street, W.C., and not to the Editor of THE HOSPITAL. Names continue to arrive in considerable numbers, but if those interested desire the Fund to be started before the end of the Jubilee Year they must issue a six-line whip to all their friends immediately.

FROM E. BEATTY, THE HOME, ST. THOMAS'S HOSPITAL.

I SHOULD very much like to become a member of the Nurses' Fund. Very few nurses seem to know anything about the proposal, or I am sure they would send in their names.

*** Nurses should read THE HOSPITAL —[ED. T. H.]

